# Open-source insect camera trap with vibrational detection and luring for monitoring *Stictocephala basalis* (Walker, Hemiptera: Membracidae: Smiliinae)

**DOI:** 10.1016/j.ohx.2024.e00604

**Published:** 2024-11-15

**Authors:** Vincent Vaughn, Andrew Ensinger, Edwin Harris, Elijah Shumway, Rachele Nieri, Vaughn Walton, John Selker, Chet Udell

**Affiliations:** a OPEnS Lab, Biological and Ecological Engineering, Oregon State University, Corvallis, OR, USA; b University of Trento, Trento, Italy

**Keywords:** Frequency analysis, Pest control, Treehoppers, Vineyard, Contact microphone, Automated system, Audio information retrieval

## Abstract

We have developed a novel device for automatic sensing, luring, and imaging insects that use substrate-borne vibrational signals for identifying and locating mating partners. The device is capable of measuring the activity patterns of these insects in a local area. It is intended to be used for monitoring pest insects; the current version of the device focuses on the treehopper species *Stictocephala basalis* (Walker, Hemiptera: Membracidae: Smiliinae) that may serve as a vector for Grapevine Red Blotch Disease. The device detects male treehoppers by sensing their mating calls using a piezoelectric contact microphone attached to a host plant, and lures them towards an imaging area by playing a prerecorded female mating call using a vibration exciter. This work is significant because previous efforts towards agricultural pest monitoring through biotremology methods has achieved only limited practical application. The trap has successfully detected and recorded wild treehopper mating calls and activity patterns, and it provides a pathway towards targeted, non-toxic pest control of various insect species that use vibrational communication. The system may be adapted to physically trap insects or alter damaging behavior in various cropping systems.

## Graphical abstract

**Figure:**
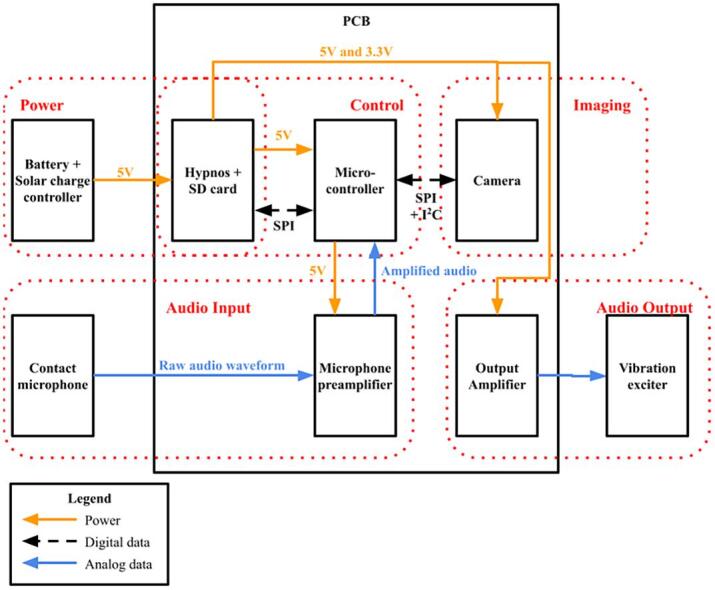

**Specifications table:** 

Hardware name	*Pied Piper*
• Subject area	• Environmental, Planetary and Agricultural Sciences
• Hardware type	• Field measurements and sensors
Closest commercial analog	AudioMoth
Open Source License	GNU GPL v3
Cost of Hardware	$230
Source File Repository	https://doi.org/10.5281/zenodo.13831951

## 1 Hardware in context

Information on the locations, times, and conditions of greatest activity of pest insects is extremely valuable to agriculture, as it can be used to develop and optimize environmentally friendly methods for controlling their populations. Additionally, such information can be used to gain a greater understanding of insect biology, plant-insect interactions, and the effect of insect activity on a wider ecosystem. This data is usually acquired by deploying insect traps throughout an environment and measuring the number of specimens that are caught over some period. The efficacy of these traps is often improved by employing lures that manipulate insect behavior through the use of semiochemicals such as synthetic pheromones, or semiophysicals like colors, lights and vibrations [1]. Some insect species do not have chemical means of communication that can be easily exploited for luring them; this is a particularly large problem with some treehopper and leafhopper species [2], which are known agricultural pests due to their ability to spread pathogens among crops that adversely affect their yields or quality [1]. This makes studying such insects in the wild difficult, as researchers must rely on inefficient and/or laborious methods that involve either manually scouting for specimens or using poorly-optimized traps employing generic stimuli. However, many insect species (including leafhoppers and treehoppers) utilize vibrational signals for communication. For this reason, farmers and researchers are increasingly turning to biotremology, the study of vibrational communication [3], to monitor and control such species (Table 1).

**Table 1:** Pied Piper power budget.

**Component**	**Current (mA)**	**Voltage (V)**	**Power consumption (mW)**	**Duty cycle (%)**	**Average power consumption (mW)**
Microcontroller + power board	42	5	210	100 %	210
Output amplifier + Vibration exciter	54	5	270	1.3 %*	4
Camera module	81	5	405	5.7 %*	23
Total average power consumption					246 mW

Battery energy capacity: 24 Wh.

**Estimated battery life (without solar power)**: 4.1 days.

*The duty cycle of these components is dependent on how many detections the trap records, which can be highly variable. The percentages shown here are estimates based on the default configuration of the device and the assumption that an average of 20 detections per day will occur. During field tests, the average number of detections recorded per day was much less than 20, meaning that the battery life shown is a low estimate.

Note: Kindly check Author Biography in pdf format (PiedPiperAuthorBios2 and PiedPiperAuthorPhotos2).

Several studies have investigated the use of vibrational signals to manipulate leafhoppers and treehoppers, which primarily consist of efforts to reduce their populations by disrupting their courting behavior [2], [4], [5]. Some theoretical and proof-of-principle work has been done with regards to luring these insects using artificially-produced mating calls, with a notable examples being a study by Polajnar J. et al.[6], which investigated the application of vibrational methods for pest control, and several investigations into vibrational traps performed by Mankin et al., who built and tested several prototype devices [7]. Korinsek et al. also developed a software architecture and detection algorithm used for detecting and luring *Aphrodes bicincta* in laboratory conditions, using a laser vibrometer and electromechanical vibration exciter to respectively measure and generate substrate vibrations [8]. Additionally, the use of vibrational signals to lure and trap insects for control of agricultural pest insects is gaining commercial success, as the BIOGARD® *Shindo Trap* relies on this principle to reduce populations of brown-marmorated stink bugs [9].

**Fig. 1:**
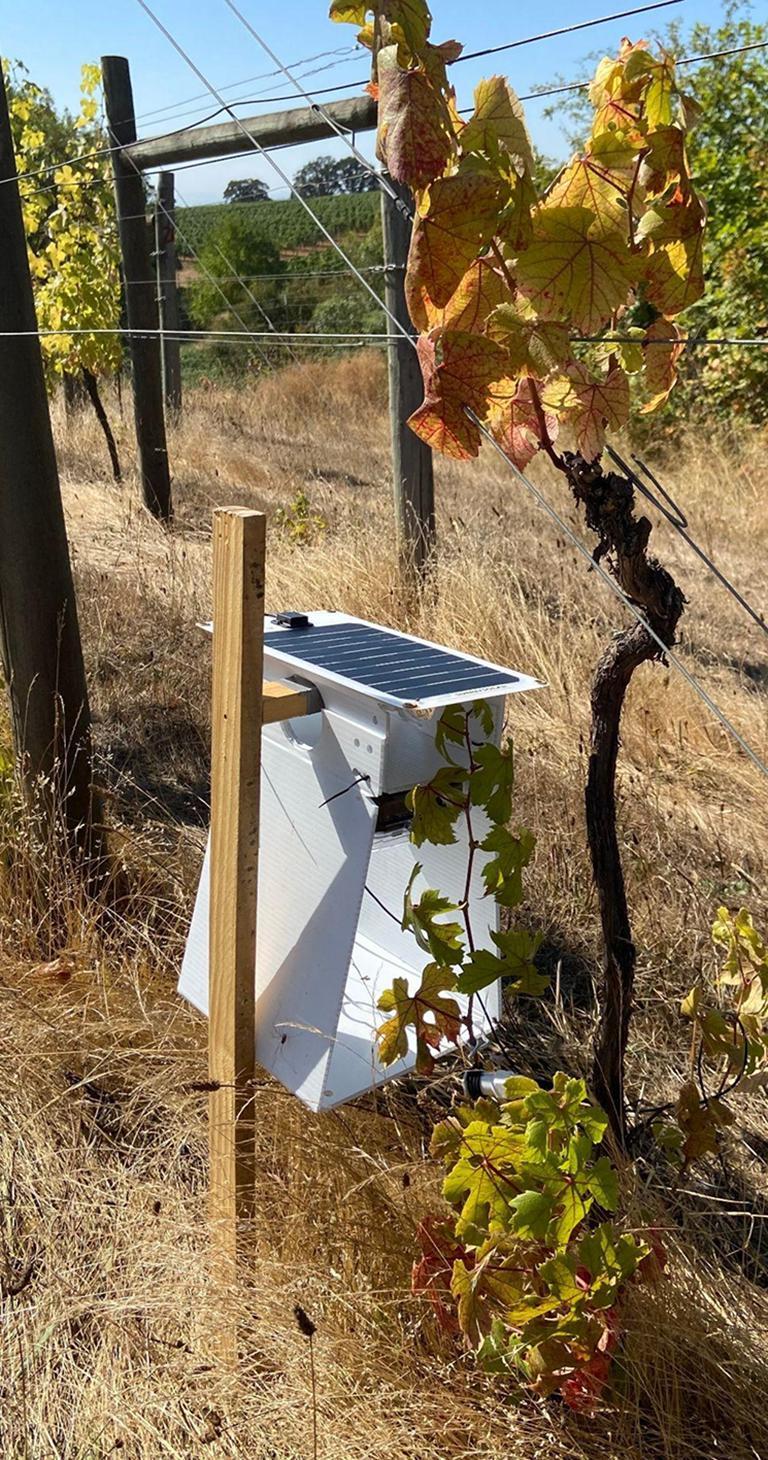
The Pied Piper *Stictocephala basalis* trap deployed on a grapevine.

There are currently no commercial devices that actively perform vibrational detection of insects, and we were unable to find a vibrational trap in the literature with hardware and software documentation sufficient for reproduction and practical application. For this reason, we have developed the Pied Piper device, a vibration detection camera trap that provides an effective, autonomous method for monitoring *S. basalis* treehoppers, (Fig. 1). The device gets its name from a popular legend of a man who lures mice from a village by playing music. We chose to target *S. basalis* due to its extensive use of vibrational communication in mating behavior similar to other species in Membracidae [10] and its significance as an agricultural pest, as this species is suspected to serve as a vector for Grapevine Red Blotch Disease [11].

Pied Piper works by detecting male treehopper mating calls using a piezoelectric contact microphone (with associated amplifier electronics, signal processing, and detection algorithm), and luring specimens to an imaging area by playing a prerecorded female mating call using a vibration exciter (Fig. 1). The trap can provide much higher-resolution temporal data on insect activity than traditional mechanical or adhesive traps, as it automatically records the exact date and time of every positive mating call detection. This allows it to provide more insight into how rapidly changing environmental conditions such as temperature, pressure, precipitation, and light can affect insect behavior and activity patterns. Additionally, the trap is sufficiently low-cost (∼$230) to allow it to be deployed at sufficient scale to measure differences in insect activity between many specific areas within a given environment.

The primary data produced by Pied Piper traps are the time, date, and substrate vibration data associated with each positive detection of a male *S. basalis* mating call. A software detection algorithm based on frequency domain analysis allows the trap to autonomously collect this data over a deployment period as long as three months, limited primarily by the availability of solar power. Pied Piper traps do not physically trap insects, as the data that would be gained from this is unnecessary given their acoustic detection capabilities. The imaging capabilities of the traps allows them to provide secondary functionality as camera traps, and is also intended to provide additional verification of acoustically detected insects in cases of ambiguous vibrational signals. As a pilot solution, not all design choices are optimal yet, but acceptable in this stage of development especially since changing the playback program according to field experience would be trivial.

The closest equivalent device to the Pied Piper trap is the AudioMoth, which is produced by Open Acoustic Devices [12] and costs $80 USD. Similarly to Pied Piper, this device serves as a platform environmental audio recording and automatic detection of audio signals [12], however it cannot measure substrate-borne vibrations and does not have playback or imaging functionality.

From here on, the substrate vibration data is referred to simply as “audio” due to its similarity to airborne vibrational data.

## 2 Hardware description

**Fig. 2:**
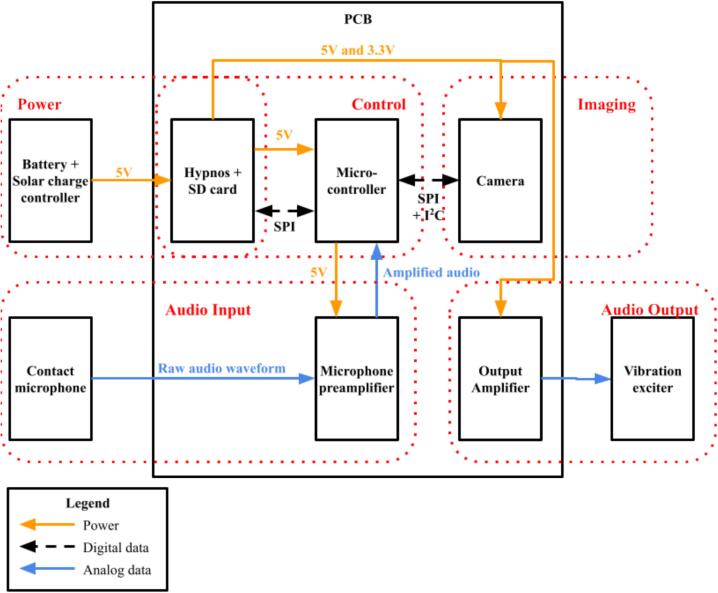
System and component breakdown of the Pied Piper trap’s electronics. Dotted outlines indicate subsystems of components based on their function, and arrows indicate connections between components.

The Pied Piper trap consists of five integrated subsystems which are each responsible for carrying out a specific function of the device. The architecture of these systems is presented in Fig. 2. These subsystems are:

1. Control: The Control system is responsible for recording audio and insect detection data, performing digital signal processing of incoming audio data from the Audio Input subsystem (particularly executing the algorithm used for detecting the presence of Treehopper mating calls), and orchestrating all the other subsystems. This system consists of a microcontroller, a power control board, and a 16 GB microSD card.
2. Power: The Power system consists of a battery and a power control board (which is shared with the Control subsystem), and it is responsible for providing power to all the other systems, as well as switching peripheral components on when they are needed. This is useful for saving power in between sample cycles.
3. Audio input: The Audio Input system is responsible for measuring, amplifying, and conditioning analog vibrational data recorded from the substrate, and sending this signal to the Control system. This system consists of a piezoelectric contact microphone, and a conditioning microphone preamplifier.
4. Audio output: This subsystem consists of a vibration exciter and a sound board, and it performs playback of prerecorded female treehopper mating calls to lure detected male treehoppers closer to the camera.
5. Imaging: The Imaging system is responsible for photographing detected male treehoppers, and it consists of a camera module and an array of LEDs to provide illumination.

All the subsystems comprising the device are connected using a custom printable circuit board (PCB). The microphone preamplifier is the only system that is directly soldered to the PCB, and the components of all other systems are connected using header pins, screw terminals, or other connectors that can be easily removed without soldering to facilitate swapping and repair of broken components. Open source components were selected where possible, and non-open source components were selected on the basis of whether they can be easily substituted. The device has a battery life of at least 4.1 days when operating at 100 % duty cycle in complete darkness, and it is capable of operating indefinitely if sufficient solar power is available.

The following subsections provide the principles of operation and detailed component breakdowns of each subsystem.

### 2.1 Control subsystem

The Control subsystem is responsible for:

● Recording and processing audio data from the microphone preamplifier and executing the mating call detection algorithm.
● Logging detection and photo data to nonvolatile storage, including the time and audio associated with each positive detection.
● Commanding the Power subsystem to provide power to components as they are needed.
● Sending audio data to the Audio Output subsystem when necessary to play back prerecorded mating calls to lure insects towards the trap.
● Commanding the Imaging subsystem to take photos.

The Control subsystem comprises the microcontroller (an Adafruit Feather M4 Express, SAMD51) and the Hypnos board, an open source PCB designed by OPEnS Lab at Oregon State University that provides power management, RTC, and data logging functionality [14]. The microcontroller is used to orchestrate all the other components of the device, and it executes the main software described in 2.1.1, 2.1.2. The Hypnos board is used to store audio data (including both the audio recorded form detections and the prerecorded mating calls played back to lure insects) and to keep track of the time and date. Additionally, the Hypnos board plays a role in the Power subsystem (detailed in section 2.2), as it is used to control power to the camera module (including illumination LEDs), vibration exciter, and SD card.

The SAMD51 Feather M4 Express was selected because the Cortex M4 SoC it is based on has both a relatively high clock speed and hardware support for floating-point arithmetic, and these are both necessary for executing the mating call detection algorithm in real time. The Hypnos V3.3 board was selected because it is directly pin-compatible with the controller, as both use the Feather formfactor.

#### 2.1.1 Software Overview

**Fig. 3:**
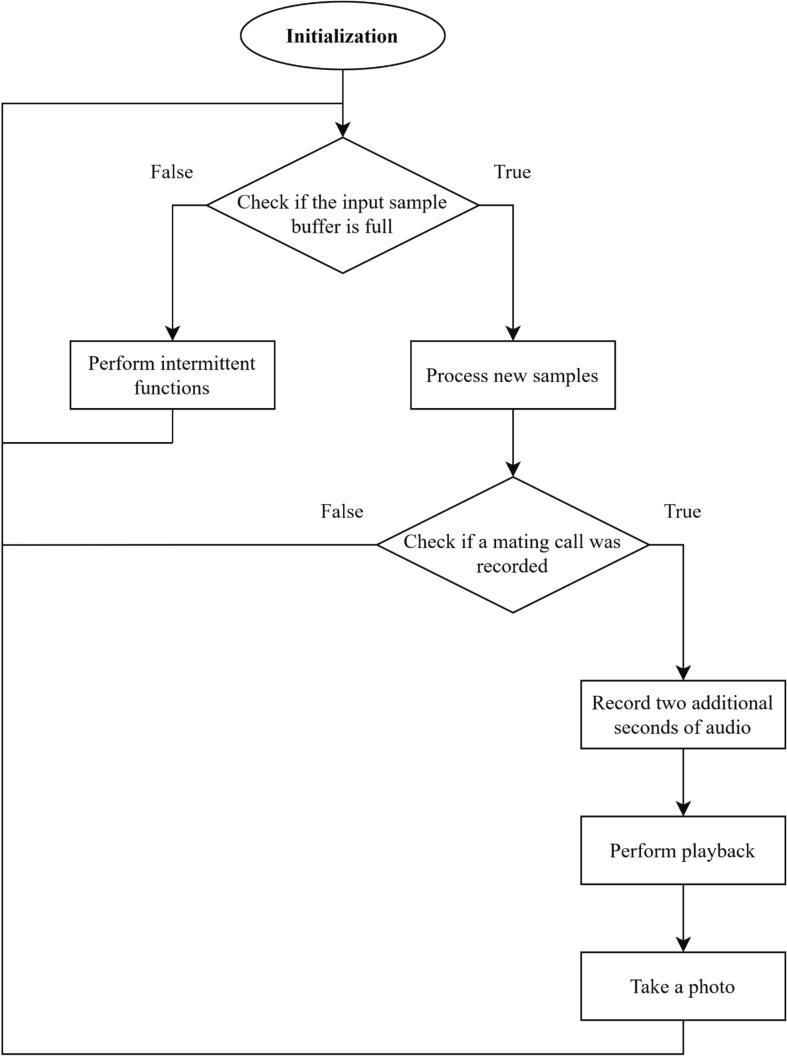
High-level state machine diagram of the Pied Piper software.

The software executed on the microcontroller is responsible for processing audio data produced by the Audio Input subsystem and controlling when specific functions of the device are to be performed. These functions include performing playback of pre-recorded luring mating calls, taking photos, and logging detection and photo data to nonvolatile storage. Fig. 3 provides a high-level state machine diagram for the Pied Piper software.

To allow mating call detections to be visually confirmed, the device is configured to immediately perform mating call playback and begin taking photos every fifteen seconds when a positive detection occurs. Additionally, the device will intermittently take photos and perform mating call playback even when no detections have recently been made to elicit a response from any treehoppers that may be present on the substrate.

The general pattern of the trap’s operation is designed to exploit the “call-fly” [10] mate searching behavior typical of male insects in the Membracidae family, in which the male periodically moves to a new plant, produces advertising calls, and eventually migrates to a new plant if no female mating calls are heard in response [10]; similar behavior has been observed in leafhoppers [13]. The trap emits artificial female mating calls in response to each successive mating call produced by the male, with the aim of forming a duet with the male and thus luring it towards the region visible to the camera. The trap will intermittently perform playback once per fifteen minutes in the absence of any positive detections, because it has also been observed that the female can initiate a duet in some species [10]. Intermittent playback is performed every fifteen minutes because this is a reasonably high-frequency rate that does not significantly impact the trap’s power budget. The duetting behavior on which the trap’s operating procedure is designed was observed in laboratory conditions when the male and female mating calls were recorded; see section 2.1.2 for further details on the setup used to capture these recordings.

#### 2.1.2 Audio signal processing and mating call detection algorithm

Based on the recording of 11 *S. basalis* males a mating call detection algorithm was designed for analyzing the incoming data from the Audio Input subsystem to determine whether a mating call has been recorded. Males were collected in the field, Yamhill county (Oregon, US). The male calls were recorded in laboratory conditions by placing males individually on a potted grapevine plant. Vibrational signals emitted by the males were recorded by means of a laser Doppler vibrometer (PDV-100, Polytec) focused on a reflective sticker attached on the plant stem and connected to a laptop through an audio interface (Celesonic US-20x20, Tascam). The recordings were digitized using the software Audacity, at 16 kHz sample rate and 32-bit depth. The algorithm is based on relatively simple frequency domain analysis. Signal processing is utilized to reject environmental noise that may interfere with it.

The mating call detection algorithm works by calculating the Fast Fourier Transform (FFT) of successive batches of samples and counting the number of windows in the main frequency buffer freqs that contain a large frequency peak within the range of a treehopper mating call. Only the magnitudes of the FFT are used; the phase information is discarded because it is highly prone to corruption by noise. The peak detection algorithm alone is prone to producing false positive detections due to transient, stochastic environmental noise. To combat this noise, the magnitude data is processed using a noise subtraction algorithm that eliminates any stochastic background.

**Fig. 4:**
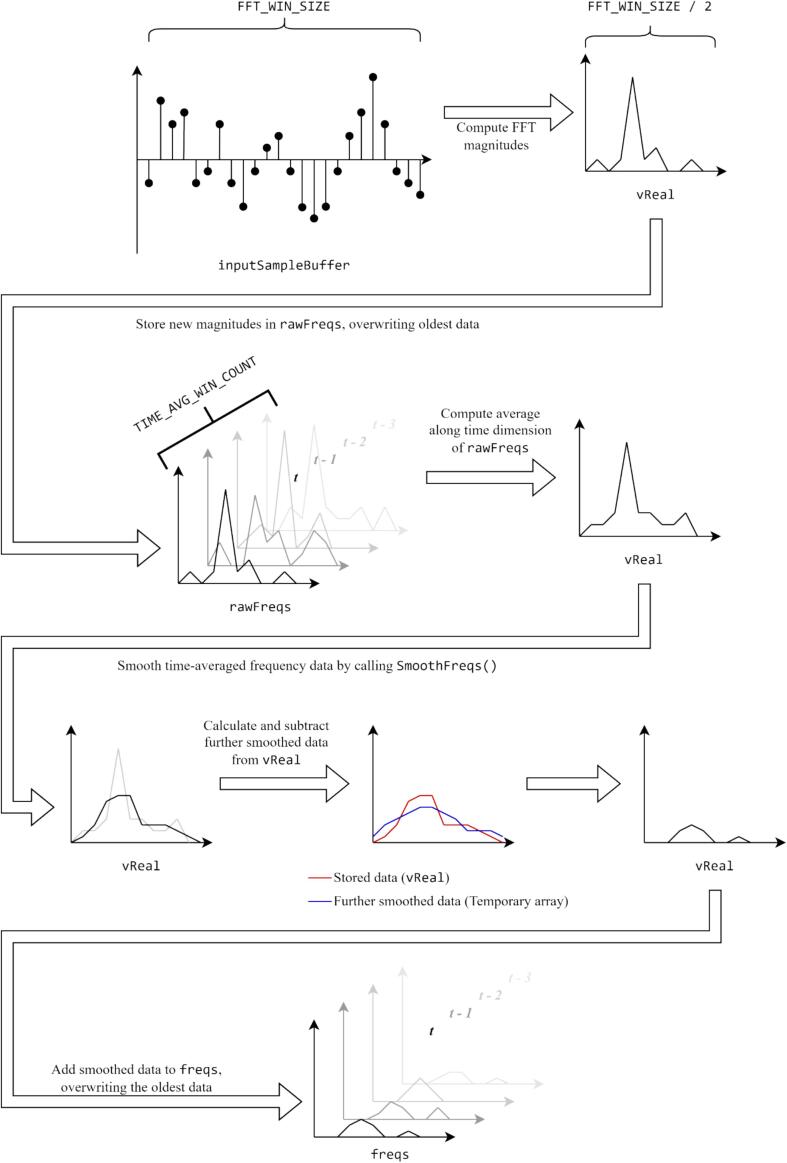
Visualization of the signal processing employed to eliminate stochastic background noise.

The mating call signals sought by the detection algorithm typically have sustained, well-defined frequencies that change slowly over time, whereas the background noise is usually stochastic and has a continuous and smooth spectral distribution over time. The noise subtraction algorithm aims to isolate any sustained, slowly-changing frequencies in the signal. To do this, it uses the following procedure (which is visualized in Fig. 4):

1. Perform time-averaging of the frequency composition of the incoming audio over several windows. This is done to extract information on the noise distribution; the sustained, sharp frequency peaks of any mating calls present will not be significantly affected by this operation, while the rapidly changing background will be smoothed.
2. Perform frequency-smoothing of the time-averaged data. This is done by convoluting the spectrum with a rectangular window, and it helps to further smooth the background noise and cause it to resemble its underlying statistical distribution.
3. Calculating a further smoothed version of the data produced by step 3 using a larger window size, and then subtracting this from the output of step 3. This step eliminates the background noise.

Treehopper mating calls can occasionally contain a series of fast, higher-frequency chirps, and they are typically removed or highly attenuated by the signal processing employed by the traps. This is inconsequential to the efficacy of the detection algorithm, as these features are too inconsistent to be useful for identifying the calls. A more sophisticated machine-learning based algorithm (such as the multilayer perceptron classifier employed by Korinsek et al. [8] to detect male *Aphrodes bicincta* ‘‘Dragonja” leafhopper mating calls) may be capable of leveraging these features to improve the performance of the software detection, however developing such an algorithm poses several challenges: implementing a robust machine learning algorithm capable of executing in real time within the constraints of a low-cost, low-power microcontroller is difficult, and training such an algorithm typically requires many samples (on the order of hundreds) to achieve good performance. The latter constraint is the primary factor currently preventing the use of a more sophisticated algorithm on this device, as we have a library of only thirteen male *S. basalis* samples, presented in the design files.

The detection algorithm and signal processing are executed every time that enough audio samples have been recorded to calculate a Fast Fourier Transform (FFT) of the incoming signal; this occurs when 256 samples have been recorded under default settings. The detection algorithm experiences no blind time because audio is sampled using an interrupt service routine (ISR) timer, which allows new audio to be recorded simultaneously while older audio is processed. To determine if a mating call is present, the algorithm scans across processed frequency data from the last 8 s of audio and tests whether it contains a sustained tone (and associated harmonics) whose frequency and duration are both within the expected ranges of a treehopper mating call.

#### 2.1.3 SD card directory structure

The microSD card installed on the Hypnos board is used to store all the data collected from the field, including images (in.jpg format) and the time, date, and audio associated with each positive detection. It is also used to store the prerecorded female mating call played back by the vibration exciter to elicit and/or respond to male mating calls. The directory structure is illustrated in the below tree, where directory names are bolded and file names are italicized:

● **DATA** − Contains all data collected during a deployment.
  - **DETS** − Contains all recorded audio data.
    ▪ *DETS.TXT* − Text file containing the time and date of each positive detection.
    ▪ 1.TXT* − Text file(s) containing the time-series audio from each positive detection.
    ▪ 2.TXT*
    ▪ …
    ▪ F1.TXT* − Text file(s) containing the spectral data from each positive detection.
    ▪ F2.TXT*
    ▪ …
  - **PHOTO** − Contains all photo data

▪ PHOTO.TXT − Text file storing the time, date, and detection number associated with each image

▪ 1.JPG* − Images taken using the camera.
▪ 2.JPG*
▪ …
  ● **PBAUD** − Stores all audio to be played back using the vibration exciter
    - *FPBX.PAD* − Female *S. basalis* mating call, stored using a special file format detailed in section 2.1.4.
  ● LOG.TXT* − Stores the value of the millisecond counter recorded periodically.

*These files are not necessary for the trap to function, but they may be present if it has been deployed before.

The playback signal stored in FPBX.PAD is a female reply signal selected among a library of mating calls recorded in laboratory conditions with the same recording set up used to record male signals. The library consisted of signals emitted by 7 different females, which all established a duet with a different male and successfully attracted the male to emitting female location.

#### 2.1.4 Playback audio file format

The audio played back by the trap is stored on the microSD card using a headerless, unsigned 16-bit (effectively 12-bit) PCM WAVE audio file with a sample rate of 4096 Hz. The samples are stored as sequential unsigned short integers, with values restricted to within the range of [0, 2^12^ –1] because the controller’s onboard DAC has 12-bit resolution. The values of the samples in the file correspond directly to the values passed to analogWrite() in the audio output interrupt service routine.

Section 5.3.2 details how to convert audio to the aforementioned format and upload it to the trap.

### 2.2 Power subsystem

The Power subsystem is comprised of the Hypnos board detailed in the above section, a 3.7 V 6600 mAh LiPo battery (Adafruit 1781), a solar charge controller (DFRobot 1712), and a 6 V 1A solar panel (DFRobot 1775). The solar charge controller is used to both charge the battery using power from the solar panel, and to provide 5 V power to the trap through a USB cable connected between itself and the controller. The battery and solar panel are used to store and generate electrical energy, respectively. The controller’s on-board 3.3 V regulator is used to convert the 5 V power provided by the solar charge controller to the 3.3 V used by itself, the SD card, and the camera’s illumination LEDs.

The controller is installed on top of the Hypnos board, and the Hypnos board is used to control power to the peripheral components connected to its associated 3.3 V and 5 V rails. The microphone preamplifier receives a constant (uncontrolled) source of both 5 V and 3.3 V power through stacking pins installed on the Hypnos board that bypass the controlled rails; this is done to allow the trap to record audio when the camera, SD card, and audio output amplifier are switched off.

### 2.3 Audio Input subsystem

The Audio Input subsystem is responsible for measuring substrate-borne vibrations and presenting these data to the Control subsystem, where it is then digitized using the microcontroller’s on-board ADC. This subsystem is comprised of a Korg piezoelectric contact microphone (CM300BK) and a microphone preamplifier (Analog Devices SSM2166).

The preamplifier is used to amplify the voltage output by the contact microphone, and it was selected due to its signal conditioning features. In particular, the preamplifier provides signal compression, which is used to allow the dynamic range (and consequently, the resolution) of the audio data to be maximized. Because the dominant frequency of treehopper mating calls is low (typically in the range of 150–200 Hz), the preamplifier is configured for optimal performance at lower frequencies. The output of the preamplifier is coupled to a voltage divider to bias the signal around half the 3.3 V logic level (which helps to prevent clipping) before being sent to analog pin A3 on the microcontroller. The raw, unbiased output of the amplifier is also available at pin A1 on the microcontroller, although this is usually not used in the software.

The contact microphone is responsible for sensing substrate vibrations and outputting this data as an AC voltage to the preamplifier. The microphone was selected because its piezoelectric element is fully enclosed in a plastic housing, which prevents it from coming into direct contact with water and sunlight while in the field; this minimizes the risk of corrosion and other weather-related degradation. The contact microphone is slightly modified: the mounting clip that the microphone normally comes with is replaced with a 3D-printed housing and an elastic band. This is because the mounting clip is best suited for attaching the microphone to flat or slightly curved surfaces rather than the plant vines, stems, and branches that the device is intended to be used with.

### 2.4 Audio Output subsystem

The Audio Output subsystem is responsible for luring insects towards the device by playing prerecorded mating calls into the substrate, and it consists of an audio amplifier breakout board (Adafruit 2130) and a vibration exciter (Dayton Audio DAEX25CT-4). The vibration exciter leads are connected directly to the output of the audio amplifier.

Upon playback, the audio amplifier is powered on using the Hypnos’ 5 V rail, and the amplifier receives audio data from the controller (pin A0) as an analog voltage signal. The audio amplifier drives the vibration exciter to transmit the vibrational signal into the substrate. A low-pass filter (consisting of a 1kΩ resistor and a 10 μF capacitor) is used between the analog output of the controller and the input of the amplifier to band-limit the audio signal and eliminate high-frequency noise. The microSD card on the Hypnos board is used to store the audio used for playback, and this audio data is read by the controller when the trap is first powered on.

### 2.5 Imaging subsystem

The Imaging subsystem consists of a camera module (Arducam Mini 2MP Plus) and a pair of surface-mount LEDs to provide illumination. This subsystem is responsible for capturing images of the substrate, and any insects present on it, when mating calls are detected and the insect is being lured. The camera module communicates with the controller through both SPI and I^2^C interfaces, and both are required for the module to function. When the camera takes an image, the data is buffered by its on-board CPLD and streamed to the controller, which records the image data directly to the microSD card on the Hypnos board. The LEDs are powered on any time the camera takes an image, and they are tied directly to the 3.3 V controlled rail of the Hypnos board; this means that they will briefly flash on any time that the SD card is powered on, as the SD card also receives power from this rail.

### 2.6 PCB

The Pied Piper PCB is used to connect all the electronic components together. The board has two layers, and it makes use of both through-hole (header pins and the terminal block) and surface-mount (everything else) components. All the components are installed on the top side of the board. The PCB has space and electrical connections for a Bluetooth module (Adafruit 2633), but this is not used in the design.

## 3 Design file summary

**Table:** 

**Design filename**	**File type**	**Open source license**	**Location of the file**
PiedPiper.ino	INO	GNU GPL v3	PiedPiper/Software/PiedPiper
PiedPiperDebug.ino	INO	GNU GPL v3	PiedPiper/Software/PiedPiperDebug
arduinoFFTFLOAT	C++ library	GNU GPL v3	PiedPiper/Software
SD card directory structure	Files	GNU GPL v3	PiedPiper/Software
AudioFileConverter.py	Python script	GNU GPL v3	PiedPiper/Software/Utilities
PiedPiperFileParser.m	MATLAB script	GNU GPL v3	PiedPiper/Software/Utilities
CameraShield.f3d	Autodesk Fusion360 design	GNU GPL v3	PiedPiper/Hardware/CAD
ImagingBox.f3d	Autodesk Fusion360 design	GNU GPL v3	PiedPiper/Hardware/CAD
MountingPlate.f3d	Autodesk Fusion360 design	GNU GPL v3	PiedPiper/Hardware/CAD
VibrationExciterLoop.f3d	Autodesk Fusion360 design	GNU GPL v3	PiedPiper/Hardware/CAD
PCB v122_2022-05-12.zip	Compressed ZIP file archive	GNU GPL v3	PiedPiper/Hardware/PCB
PiedPiperPCB.brd	Autodesk EAGLE PCB design	GNU GPL v3	PiedPiper/Hardware/PCB
PiedPiperPCB.sch	Autodesk EAGLE electrical schematic	GNU GPL v3	PiedPiper/Hardware/PCB
Male_[…].wav	Male S. basalis mating call recordings	GNU GPL v3	PiedPiper/Data
Female.wav	Female S. basalis mating call recording	GNU GPL v3	PiedPiper/Data

All design files are located the following Zenodo Reposiatory: https://doi.org/10.5281/zenodo.13831951.

**PiedPiper.ino**: Main arduino program that executes on the controller. Requires piedPiper.h, piedPiper.cpp, and arduinoFFTFloat to compile and function properly.

**PiedPiperDebug.ino**: Alternative version of PiedPiper.ino intended for independently testing various hardware and software subsystems. This version should not be used when the devices are deployed to collect data in the field. Requires piedPiper.h, piedPiper.cpp, and arduinoFFTFloat to compile and function properly.

**arduinoFFTFLOAT**: Modified version of arduinoFFT, a library used to compute Fast Fourier Transforms on Arduino. The library has been modified to exclusively use single-precision floating point arithmetic.

**SD card directory structure**: The files contained within this file should be copied into the SD card installed into the Hypnos board. This file contains the recording of a female treehopper mating call played back by the vibration exciter within a processed audio file FPBX.PAD.

**AudioFileConverter.py**: Python script used to convert a 4096 Hz, 16-bit unsigned PCM WAVE audio file into the special format used for the vibration exciter playback. Requires Python version 3 or greater.

**PiedPiperFileParser.m**: MATLAB script used to parse data collected by traps.

**CameraShield.f3d**: Autodesk Fusion design file of the camera shroud. An STL file of this component is available in PiedPiper/Hardware/CAD/STL.

**ImagingBox.f3d**: Autodesk Fusion design file of the imaging box. A DXF file of the flat pattern used to make the box is available in PiedPiper/Hardware/CAD/DXF.

**MountingPlate.f3d**: Autodesk Fusion design file of the backplate. An STL file of this component is available in PiedPiper/Hardware/CAD/STL.

**VibrationExciterLoop.f3d**: Autodesk Fusion design file of the vibration exciter loop. An STL file of this component is available in PiedPiper/Hardware/CAD/STL.

**PCB v122_2022-05**–**12.zip**: ZIP file containing the Gerber files of the Pied Piper PCB.

**PiedPiperPCB.brd**: Autodesk EAGLE design file containing the Pied Piepr PCB design.

**PiedPiperPCB.sch**: Autodesk EAGLE design file containing the schematic of the PCB.

**Male_[**…**].wav**: Thirteen vibrometer recordings of male *S. basalis* mating calls recorded using a Polytec PDV-100 laser vibrometer.

**Female.wav**: Female *S. basalis* mating call recorded using a Polytec PDV-100 laser vibrometer.

## 4 Bill of materials summary

Throughout the bill of materials provided below, “Cost” refers to the total cost contributed by each component to the overall cost of constructing a single device.

Mechanical components

**Table:** 

**Designator**	**Component**	**Qty.**	**Cost**	**Unit cost**	**Source**	**Material**
Case	Pelican 1040 Micro (Clear)	1	27.95	27.95	https://www.amazon.com/gp/product/B001GGBORU	Plastic
Cable gland	PG7 Cable gland	1	0.40	0.40	https://www.amazon.com/gp/product/B06Y5HGYK2	Nylon
M2.5 Screw (PCB), M2.5 Screw (Camera)	M2.5 × 4.5 mm	6	0.96	0.16	https://www.digikey.com/en/products/detail/essentra-components/50M025045H005/11638179	Nylon
M2 Screw (Amplifier)	M2 × 4 mm Screw	2	0.32	0.16	https://www.digikey.com/en/products/detail/essentra-components/50M020040P005/11639294	Nylon
M3 Screw (Charge controller)	M3 × 6 mm Screw	2	0.16	0.08	https://www.amazon.com/gp/product/B071XPTK7Z	Nylon
Nut (PCB), Nut (Camera)	M2.5 Nut	6	0.72	0.12	https://www.digikey.com/en/products/detail/essentra-components/04M025045HN/9677099	Nylon
M2 Nut (Amplifier)	M2 Nut	2	0.30	0.15	https://www.digikey.com/en/products/detail/essentra-components/04M020040HN/9677098	Nylon
Nut (Charge controller)	M3 Nut	2	0.12	0.06	https://www.amazon.com/gp/product/B01IWUSDYY	Steel
Standoff (Camera)	M2.5 × 5 mm Standoff	2	0.58	0.29	https://www.digikey.com/en/products/detail/assmann-wsw-components/V6622A/3511496	Brass
Standoff (Amplifier)	M2 × 8 mm Standoff	2	0.58	0.29	https://www.digikey.com/en/products/detail/würth-elektronik/97https://doi.org/1080244/9488615	Brass
Standoff (Charge controller)	M3 × 25 mm Standoff	2	0.20	0.10	https://www.amazon.com/gp/product/B083GNJMXZ	Nylon
Elastic band	Elastic band	1	0.02	0.02	https://www.amazon.com/gp/product/B000BLJEKU	Rubber
Desiccant	5 g silica gel desiccant pack	1	0.16	0.16	https://www.amazon.com/gp/product/B08YJH2J6Q	Composite/Other
Zip ties	POWER FIRST Cable Tie: 4 in Nominal Lg, 1 in Nominal Max. Bundle Dia., 0.1 in Wd, Black, 21	4	4.80	0.048	https://www.grainger.com/product/POWER-FIRST-Cable-Tie-4-in-Nominal-Lg-36J128	Polymer

The contact microphone mounting piece, vibration exciter loop, and camera shroud are 3D printed using the ASA filament. The backplate and imaging box are laser-cut from the corrugated plastic.

Electrical components

**Table:** 

**Designator**	**Component**	**Qty.**	**Cost**	**Unit cost**	**Source**	**Material**
Feather	Adafruit Feather M4 Express	1	22.95	22.95	https://www.adafruit.com/product/3857	Composite
Hypnos	OPEnS Hypnos V3.3	1	27.00	27.00	https://github.com/OPEnSLab-OSU/OPEnS-Hypnos/tree/master/Hypnos%20V3.3	Composite
Coin cell battery	Energizer CR1220 Low Drain 3 V lithium Battery	1	1.35	1.35	https://www.amazon.com/gp/product/B003CU3E2Q	Non-specific
PCB	Pied Piper V3 PCB	1	3.26	3.26	https://www.pcbway.com	Composite
SD card	SanDisk 32 GB microSDHC card	1	5.58	5.58	https://www.amazon.com/gp/product/B08J4HJ98L	Non-specific
Camera module	Arducam Mini 2MP + OV5260	1	25.99	25.99	https://www.amazon.com/gp/product/B012UXNDOY	Non-specific
Amplifier	Adafruit Mono 2.5 W Class D Audio Amplifier	1	3.95	3.95	https://www.adafruit.com/product/2130	Non-specific
Vibration exciter	Dayton Audio DAE25CT-4	1	14.02	14.02	https://www.amazon.com/gp/product/B00M292316	Non-specific
Charge controller	DFR0559	1	7.90	7.90	https://www.digikey.com/en/products/detail/dfrobot/DFR0559/9356334	Non-specific
Solar panel	Semi Flexible Monocrystalline Solar Panel (6 V 1A)	1	17.50	17.50	https://www.dfrobot.com/product-1775.html	Non-specific
Battery	Lithium Ion Battery Pack – 3.7 V 6600 mAh	1	24.50	24.50	https://www.adafruit.com/product/353	Non-specific
Microphone	Korg Tuner (CM300WHBK)	1	14.98	14.98	https://www.amazon.com/gp/product/B07DZVYFC1	Non-specific
MicroUSB cable	Short (7″) MicroUSB Cable	1	1.80	1.80	https://www.amazon.com/gp/product/B01FSYBQ9Q	Non-specific
Male header pins	Break-away 0.1″ 36-pin strip male header	2	1.00	0.50	https://www.adafruit.com/product/392	Non-specific
Female header pins	36-pin 0.1″ Female header	2	1.00	0.59	https://www.adafruit.com/product/598	Non-specific
Short male header pins	Male Header 36-pin 0.1″ Short Break-away Type	1	0.50	0.50	https://www.adafruit.com/product/3009	Non-specific
Short female header pins	36-pin 0.1″ Short Female Header	1	1.60	1.60	https://www.adafruit.com/product/3008	Non-specific
Feather stacking header pins	Stacking Headers for Feather – 12-pin and 16-pin female headers	1	1.25	1.25	https://www.adafruit.com/product/2830	Non-specific
Right-angle female header pins	0.1″ 36-pin Strip Right-Angle Female/Socket Header	1	0.99	0.99	https://www.adafruit.com/product/1542	Non-specific
Terminal block	uxcell 20 Pcs 3.5 mm Pitch 2Pin PCB Mount Screw Terminal Block Connector	1	0.30	0.30	https://www.amazon.com/gp/product/B00R1M0CBM	Non-specific
Preamplifier	SSM2166S	1	9.25	9.25	https://www.digikey.com/en/products/detail/analog-devices-inc/SSM2166SZ/654310	Non-specific
LED1	MP-3030-120H-50-80	1	0.29	0.29	https://www.digikey.com/en/products/detail/luminus-devices-inc/MP-3030-120H-50-80/14301411	Non-specific
LED2	MP-3030-120H-40-80	1	0.29	0.29	https://www.digikey.com/en/products/detail/luminus-devices-inc/MP-3030-120H-40-80/14301416	Non-specific
D1	3.3 V TVS S32-SOD-323	1	0.40	0.40	https://www.digikey.com/en/products/detail/eaton-electronics-division/STS321033B100/13280517	Non-specific
R1	1 MΩ SMD resistor, pkg: 1206	1	0.10	0.10	https://www.digikey.com/en/products/detail/walsin-technology-corporation/WR12X105-JTL/13240641	Non-specific
R2, R5	20 kΩ SMD resistor, pkg: 1206	2	0.20	0.10	https://www.digikey.com/en/products/detail/stackpole-electronics-inc/RNCP1206FTD20K0/2240380	Non-specific
R3, R7, R8	10 kΩ SMD resistor, pkg: 1206	3	0.30	0.10	https://www.digikey.com/en/products/detail/stackpole-electronics-inc/RNCP1206FTD10K0/2240370	Non-specific
R4	1.1 kΩ SMD resistor, pkg: 1206	1	0.10	0.10	https://www.digikey.com/en/products/detail/stackpole-electronics-inc/RNCP1206FTD1K10/2240338	Non-specific
R6	40 kΩ SMD resistor, pkg: 1206	1	0.36	0.63	https://www.digikey.com/en/products/detail/yageo/RT1206BRD0740KL/5936957	Non-specific
R9	1 kΩ SMD resistor, pkg: 1206	1	0.10	0.10	https://www.digikey.com/en/products/detail/stackpole-electronics-inc/RNCP1206FTD1K00/2240337	Non-specific
R10, R11	5.6 Ω SMD resistor, pkg: 1206	2	0.20	0.10	https://www.digikey.com/en/products/detail/stackpole-electronics-inc/RMCF1206FT5R60/1759510	Non-specific
C1, C6	10 μF SMD capacitor, pkg: 0805	2	0.20	0.10	https://www.digikey.com/en/products/detail/samsung-electro-mechanics/CL21A106KOQNNNG/3894417	Non-specific
C2	3.3 μF SMD capacitor, pkg: 0805	1	0.21	0.21	https://www.digikey.com/en/products/detail/samsung-electro-mechanics/CL21A335KPFNNNG/3894437	Non-specific
C3, C7	1 μF SMD capacitor, pkg: 0805	2	0.20	0.10	https://www.digikey.com/en/products/detail/samsung-electro-mechanics/CL21B105KAFNNNE/3886724	Non-specific
C4	10 nF SMD capacitor, pkg: 0805	1	0.10	0.10	https://www.digikey.com/en/products/detail/samsung-electro-mechanics/CL21B103KBANNNC/3886673	Non-specific
C5	2.2 μF SMD capacitor, pkg: 0805	1	0.12	0.12	https://www.digikey.com/en/products/detail/samsung-electro-mechanics/CL21B225KOFNNNE/3886820	Non-specific

Bulk Materials

**Table:** 

**Designator**	**Component**	**Qty.**	**Cost**	**Unit cost**	**Source**	**Material**
Solder	Solder Wire Lead Free Rosin Core Flux 0.8 mm	5 g	0.80	0.16 / g	https://www.amazon.com/gp/product/B07Q167J98	Metal
Wire	18 AWG pre-tinned copper wire	0.4 m	0.20	0.5 / m	https://www.amazon.com/dp/B09BFF8CX7	Metal
Heat-shrink tubing	Heat-shrink tubing strips, assorted sizes.	0.01 m	0.40	4.02 / m	https://www.amazon.com/dp/B07WWWPR2X	Polymer
Solder paste	Chip Quik SMDLTLFP Solder Paste	0.5 mL	1.60	3.19 / mL	https://www.amazon.com/dp/B0195V1QEI	Non-specific
Acrylic sheet*	0.125″ x 12″ x 12″ acrylic sheet	∼19 in^2^	0.74	0.04	https://www.grainger.com/product/1UNK4	Polymer
Corrugated plastic*	White Polypropylene Corrugated Pad	∼352 in^2^	3.30	0.01	https://www.grainger.com/product/GRAINGER-APPROVED-Corrugated-Pads-24-in-Wd-56EC59	Polymer
3D-printer filament*	White ASA 3D printer filament, 1.75 mm dia.	∼60 g	2.40	0.04 / g	https://www.3dxtech.com/product/3dxmax-asa/	Polymer
Vibration exciter wire	Silver Aluminum Wire	1 m	1.10	1.1 / m	https://www.amazon.com/dp/B07HHYPXH2	Metal
Epoxy	GORILLA Epoxy Adhesive	∼2 cc	0.82	0.41 / cc	https://www.grainger.com/product/4YKT2	Polymer
Hook-and-loop tape	VELCRO Brand Heavy Duty Tape with Adhesive	4 in	0.44	0.11 / in	https://www.amazon.com/dp/B00006RSP1	Non-specific

## 5 Build instructions

This section details the process for building the Pied Piper device. Each subsection herein contains the procedure for building a specific component of the device, as well as information on specific safety precautions, tools, materials, and processes used in each procedure. Every build procedure must be performed in a well-ventilated area and all technicians must wear safety glasses, as every procedure involves the use of soldering and/or application of adhesives or surface coatings that may produce harmful volatile substances. The instructions in each subsection can be completed separately from all the others, and there is no specific order in which each procedure must be completed (except for 5.3, which must be performed last). This is intended to make the construction of the device as time efficient as possible by allowing work to be done on different components of the device simultaneously, particularly when adhesives or coatings are drying. The device can be built with approximately six hours of work in a single session, although at least 24 h will need to pass before the unit can be deployed to allow adhesives and conformal coatings to fully dry.

It is recommended to have every 3D-printed component of the device fabricated before beginning the build procedure. There are 4 total parts that must be printed, and the settings that should be used for printing them are:

**Table:** 

• 0.2 mm primary layer height	● Support materials for overhangs enabled
● 60° overhang threshold	● ASA (or some other weather-resistant plastic) filament
● 50 % infill	

The file “allParts.stl” in the Zenodo repository contains all four parts in a pre-arranged layout that is optimal for use in an FDM printer.

### 5.1 Electronics

This section details the process for building all the electronics for the Pied Piper device, including the PCB (and associated components), camera module, and contact microphone. Hand soldering of wires, surface-mount components, and through-hole components occurs frequently in these procedures; as such, there are associated safety hazards, including a risk of burns or fire from the use of a soldering iron or heat gun, and a risk of exposure to lead from the solder (although we strongly recommend the use of lead-free solder). Additionally, there is a risk of respiratory irritation and other hazards when conformal coating is applied to components. To avoid these risks, a well-ventilated workspace should be used throughout every procedure, and the workspace must be thoroughly cleaned both before and after use. Proper soldering technique and safety precautions should be exercised to minimize the risk of fire or burns. Refer to and follow safety instructions provided by the manufacturers of all equipment and materials used throughout each build procedure.

Throughout the following procedures, it is assumed that the reader has a basic understanding of soldering and SMD reflow operations.

#### 5.1.1 PCB

This section contains the procedure for building the PCB, microcontroller, audio board, and power board. Most of this procedure consists of soldering of both SMD and through-hole components, and applying conformal coating.

**Table:** 

**Materials used**	
Solder paste	Silicone conformal coating
Microcontroller (+pins)	PCB
Audio board (+pins)	Hypnos board (+pins)
Female header pins	Male header pins
SSM2166	Capacitors, resistors
Isopropyl alcohol	
**Tools used**	
IC heater OR temperature-controlled heat gun	Soldering iron

1. Prepare a syringe of low-temperature solder paste in accordance with the manufacturer’s instructions.
2. Apply solder paste to every SMD pad on the Pied Piper PCB. These include the pads for R1 through R5, C1 through C6, and the SSM2166 amplifier. Apply only enough paste to cover each pad with a thin layer. Fig. 5, shown below, provides an example of an acceptable application of solder paste:

**Fig. 5:**
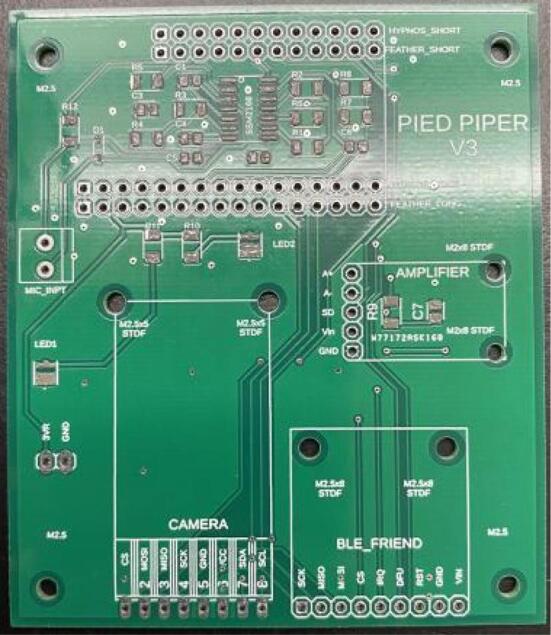
Pied Piper PCB with solder paste applied.
3. Place all the SMD components on the PCB in the following sequence: C4, R1, C1, R2, C6, C3, SSM2166, C2, R3, C5, R4, R5. Ensure that each component is well-adhered to the paste, and that the paste has not bridged any pads together (Fig. 6).

**Fig. 6:**
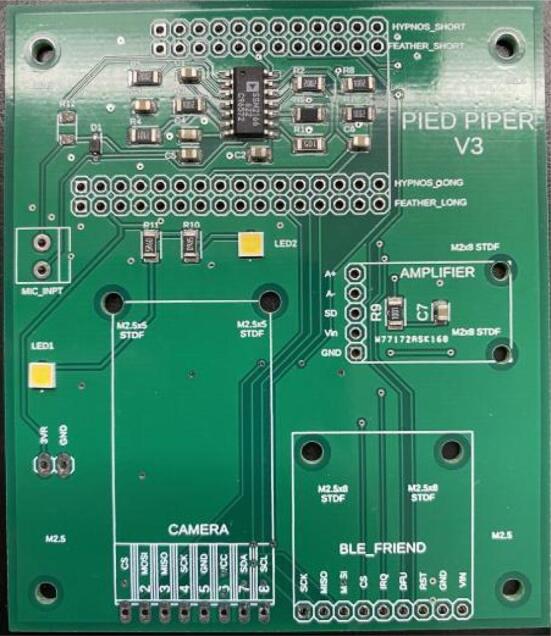
Example of SMD component application to the Pied Piper PCB prior to reflow.
4. Solder the components to the PCB. We recommend using a reflow oven to do this, if available. Place the PCB into the reflow oven, and refer to the instructions provided by the manufacturer of the oven to select and execute the thermal profile required for the solder paste.
5. Closely inspect the PCB to ensure that the soldering was successful. All the solder joints should appear to be silvery rather than matte gray, and none of the pads should be shorted together by solder (Fig. 7)..

**Fig. 7:**
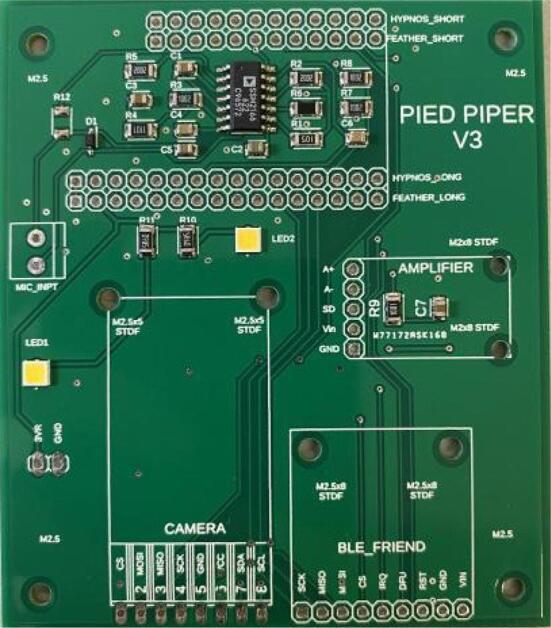
The Pied Piper PCB after SMD components have been soldered.
6. Populate the through-hole components. Solder the terminal block to the board first, then solder rows of 2.54 mm-pitch female header pins, then install standoffs (with associated nuts) last. The headers referenced in the bill of materials are all 36 pins long, and they will need to be trimmed to the correct sizes. When trimming headers, do not cut along the edge of the final pin that will be soldered to the board; always cut along the center of a “sacrificial” pin after it, to ensure that the final used pin will not be damaged. Use short headers for the amplifier contacts, and 90-degree headers for the camera module contacts (Fig. 8).

**Fig. 8:**
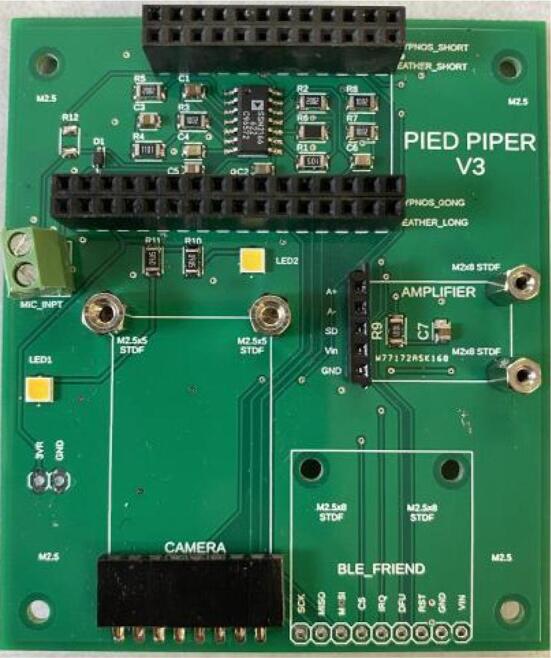
The Pied Piper PCB after installation of female header pins.
7. Solder full-size male header pins to the microcontroller, and short male header pins to the amplifier. Solder rows of stacking pins and male header pins to the power control board. The stacking pins must be oriented such that the female headers are on the populated side of the power board, and the male pins must have their long ends on the opposite side.
8. Lay out the PCB, controller, camera module, amplifier, and Hypnos board over a disposable surface. Using a brush, apply a thin layer of conformal coating to both sides of each component, while being careful to avoid getting the coating onto any electrical contacts (such as the header pins, USB ports, and microSD card slot). **Perform this step in a very well-ventilated area**. Allow the coating to dry for at least 24 h before proceeding to the next step.
9. Install the controller, camera module, amplifier, and Hypnos board onto the PCB as shown. Use M2 screws to secure the amplifier and M2.5 screws to secure the camera module (Fig. 9).

**Fig. 9:**
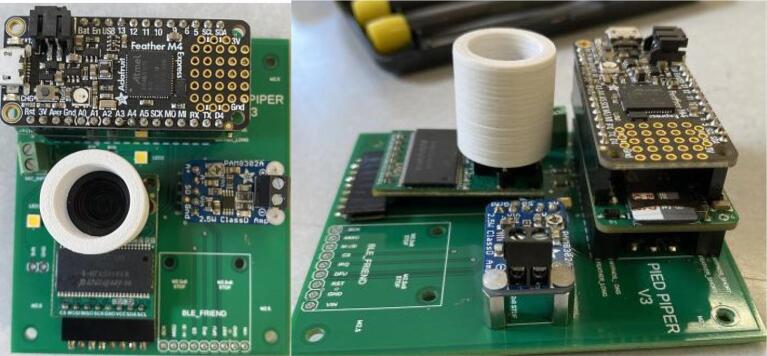
The Pied Piper PCB after camera, amplifier, and Hypnos board are installed.

#### 5.1.2 Contact microphone

This subsection provides the build procedure for the contact microphone used for sensing treehopper mating calls.

**Table:** 

**Materials used**	
Solder	Silicone conformal coating
Flux	Large heat shrink tubing
Contact microphone	Small heat shrink tubing
Epoxy adhesive	Contact microphone housing
**Tools used**	
Heat gun	Soldering iron
Side cutters	Wire strippers

1. Remove the connector from the contact microphone using a pair of wire cutters, and remove approximately 2 cm of insulation from the end of the wire using wire strippers (Fig. 10).

**Fig. 10:**
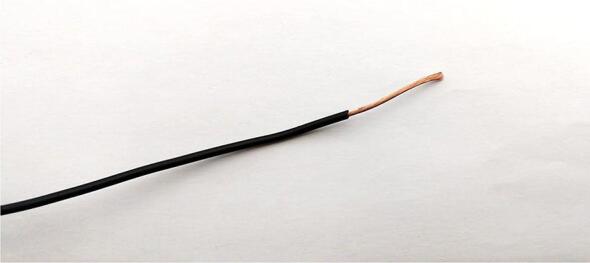
Exposed end of the contact microphone wire after removing the connector and stripping off 2 cm of insulation.
2. Remove the clip mechanism from the contact microphone using side cutters. Avoid damaging the soft rubber contact surface of the microphone (Fig. 11).

**Fig. 11:**
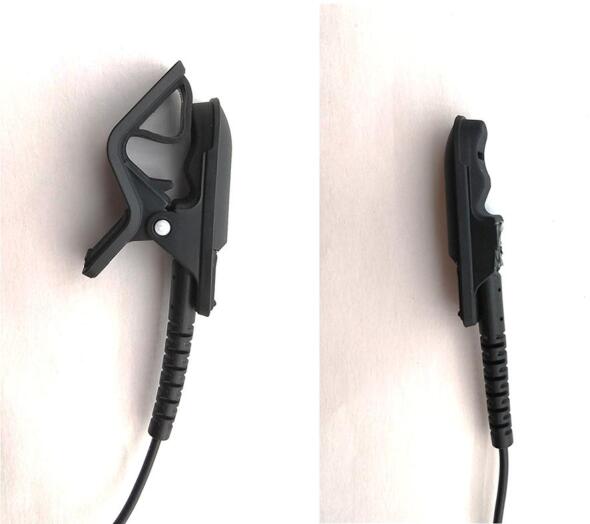
Before and after removing the mounting clip from the contact microphone.
3. Carefully untwist the exposed outer braided copper layer at the exposed end of the wire, and pull the central wire out to the side. Twist the outer copper back together separately from the inner wire. Strip 1 cm of insulation off the end of the central wire, and twist the newly exposed copper to prevent it from becoming frayed (Fig. 12)

**Fig. 12:**
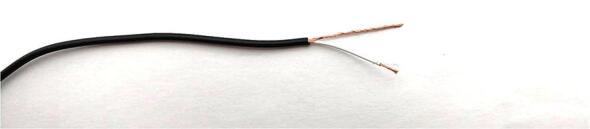
Exposed end of the contact microphone wire after separating the inner and outer conductors.
4. Using a soldering iron, apply solder to the exposed copper of both the outer and inner conductors, such that they form solid conductors that will not untwist. Slip a small piece of heat shrink tubing onto the end of the outer conductor that is long enough to shield most of the exposed copper, and shrink this tubing using a heat gun (Fig. 13)

**Fig. 13:**
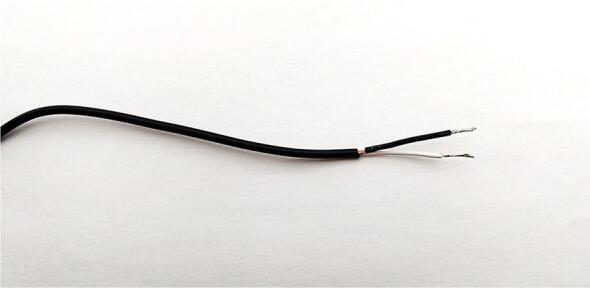
Exposed end of the contact microphone wire after both of the conductors have been soldered, and the outer conductor has been insulated with heat shrink tubing.
5. Slip a small piece of heat-shrink tubing onto the contact microphone cable. Line this tubing up with the exposed end of the cable such that it covers approximately 5 mm of the exposed conductor connecting to the outer layer of braided copper in the cable. Using a heat gun, heat the tubing such that shrinks and becomes secured to this position
6. Move the previously applied thin segment of heat shrink tubing to a position approximately 10 cm from the end of the cable. Using a heat gun, heat the tubing such that shrinks and becomes secured to this position
7. Position the previously applied wide segment of heat shrink tubing to the same position as the tubing that was heated in the previous step. Using a heat gun, heat the tubing such that shrinks and becomes secured to this position
8. Prepare a small amount of epoxy adhesive in accordance with the manufacturer’s instructions. Apply a line of epoxy to the inside of the contact microphone mounting component and adhere the contact microphone to it.
9. Allow the epoxy to dry for at least 24 h (Fig. 14)

**Fig. 14:**
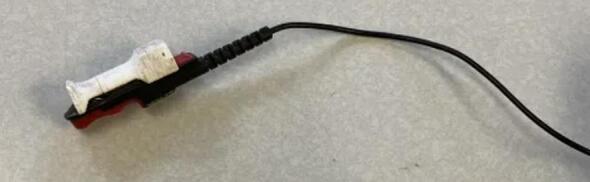
contact microphone completed with 3D printed attachment.

### 5.2 Case assembly

**Table:** 

**Materials used**	
Case	Large heat shrink tubing
Cable gland	Small heat shrink tubing
Assembled PCB	Backplate
Epoxy adhesive	
**Tools used**	
Heat gun	Side cutters
Soldering iron	Wire strippers

1. Use a small amount of epoxy adhesive to affix the vibration exciter loop to the outside of the case, and drill a 13–15 mm hole in the top of the case in the approximate positions shown (Fig. 15).

**Fig. 15:**
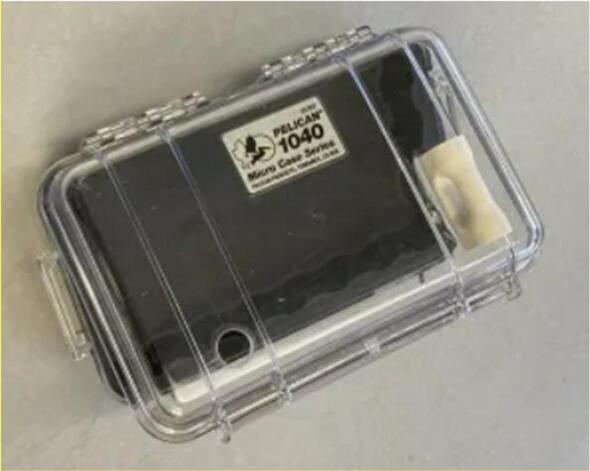
Case with 13–15 mm hole location shown.
2. Wait 24 h for the epoxy to cure. Install a cable gland in the hole and thread the solar panel and contact microphone leads through the gland. Place a large piece of heat-shrink tubing over both the solar panel and microphone leads at the position where the cable gland will close, and heat the tubing using a heat gun (Fig. 16).

**Fig. 16:**
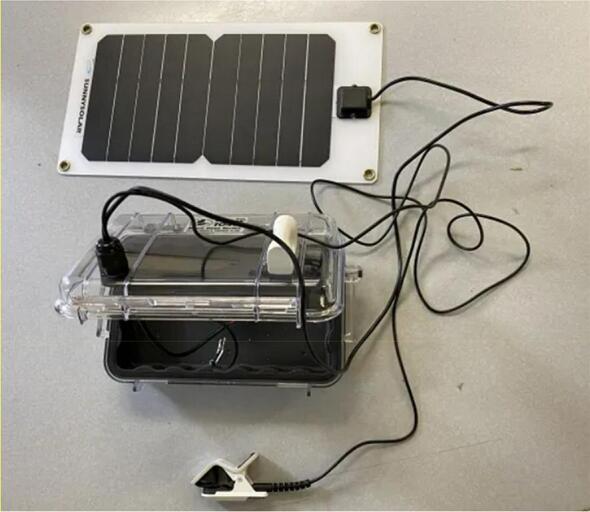
Case with gland installed in hole.
3. Affix the PCB to the laser-cut backplate using M2.5 screws and nuts. Additionally, affix two 25 mm M3 standoffs in the positions shown to support the solar charge controller (Fig. 17).

**Fig. 17:**
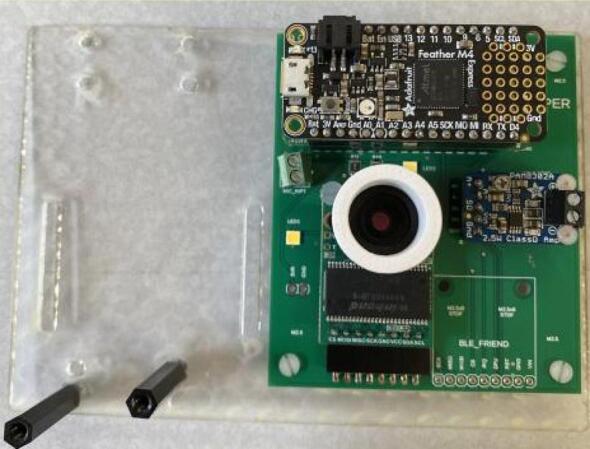
Case with gland installed in hole.
4. Feed a cable tie along the back of the backplate and through the slots as shown. Solder leads onto the vibration exciter using 18 gauge wire and affix it to the inside surface of the case lid immediately underneath the vibration exciter loop. Insert the PCB and backplate assembly into the case (Fig. 18).

**Fig. 18:**
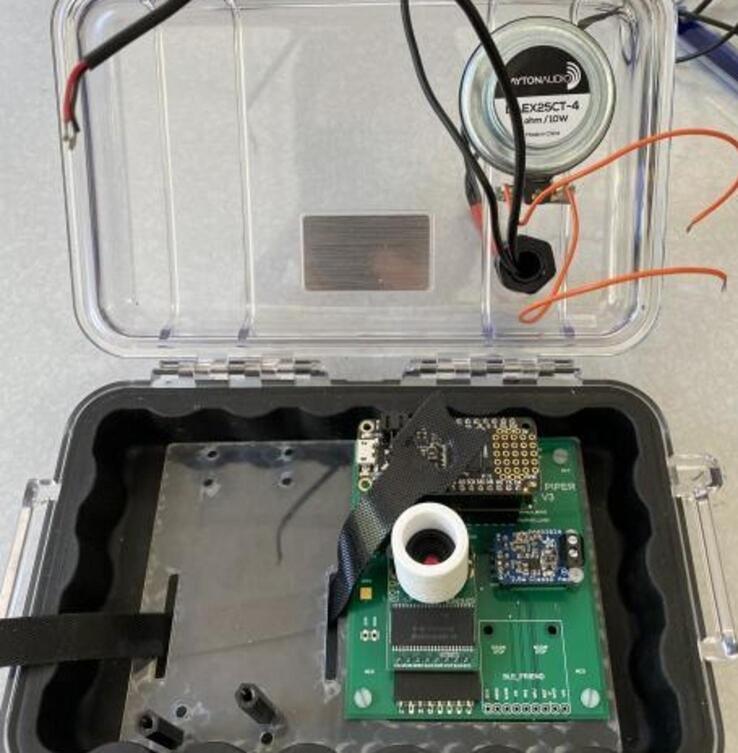
shows wire installation.
5. Connect the microphone leads to the terminal block on the PCB, the vibration exciter leads to the terminal block on the output amplifier, and place a battery between the slots in the backplate. Tightly secure the battery using the cable tie (Fig. 19).

**Fig. 19:**
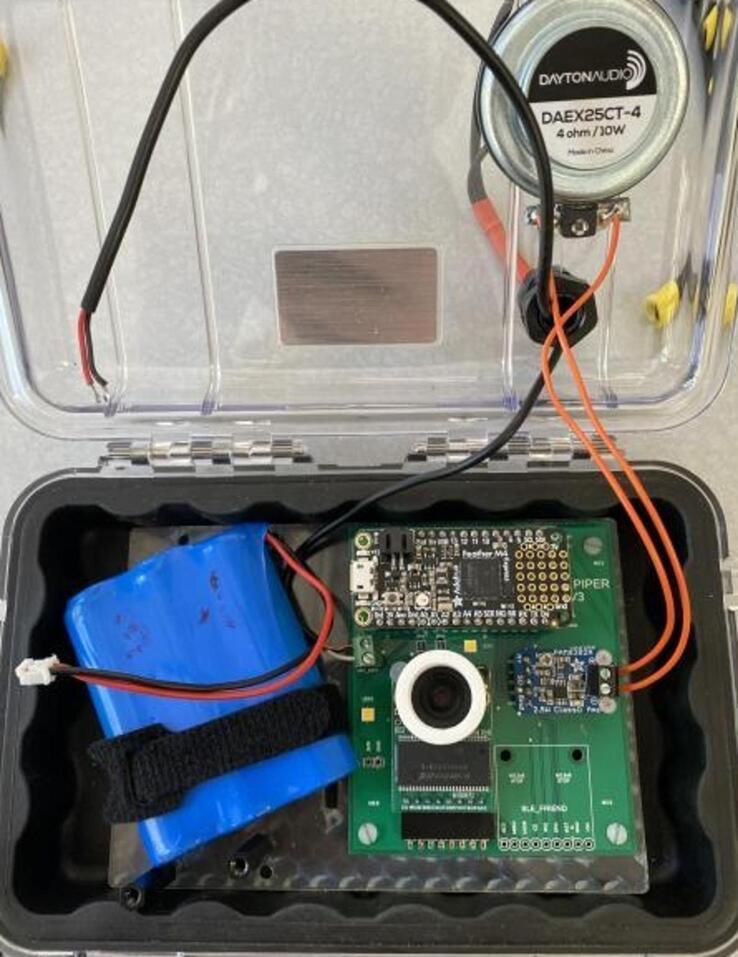
shows microphone leads.
6. Install the solar charge controller on the standoffs using the designated M3 screws. Insert the battery and solar panel leads into the terminals shown below. Connect a microUSB cable between the controller and solar charge controller. Close the case, being careful to avoid pinching wires (Fig. 20).

**Fig. 20:**
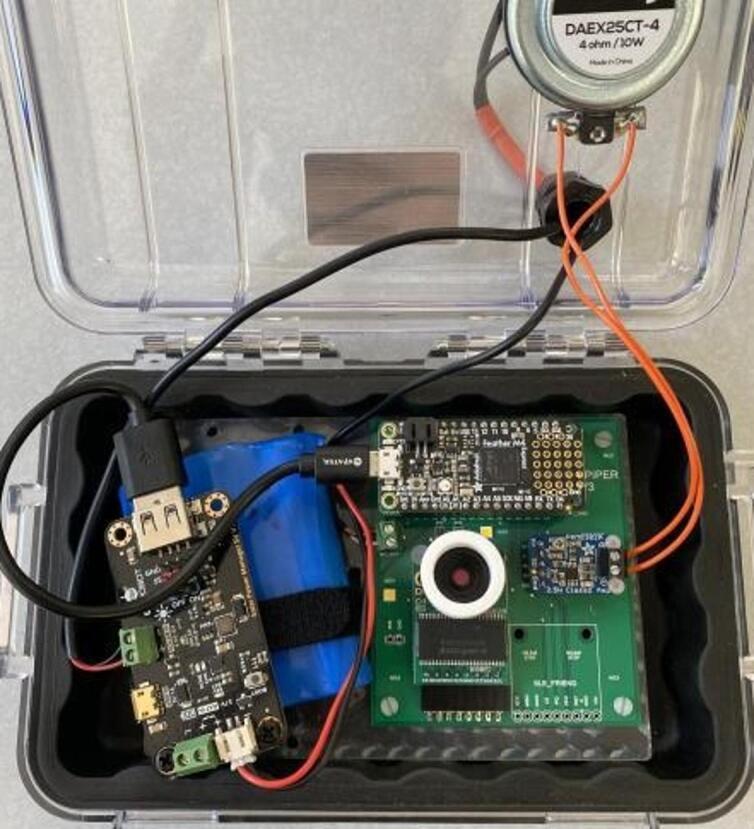
**S**hows added solar charge controller.

### 5.3 Software upload

This section describes the process used to upload software and audio data to the trap.

#### 5.3.1 microSD card formatting

1. Insert the microSD card into a computer capable of formatting external drives, using an adapter if necessary.
2. Format the card with a single 32 GB FAT32 partition.
3. Copy the contents of the “Software/SD card directory structure” folder onto the card.
4. Safely eject the card and insert it into the Hypnos board.

#### 5.3.2 Playback audio upload

This section outlines the procedure for formatting and uploading playback audio to the trap. These steps are only necessary if the trap is being deployed for a species other than S. basalis.

1. Acquire a sample of audio to be played back by the trap. This sample must be no more than eight seconds in duration, and cannot have significant spectral energy above 2048 Hz.
2. Using audio editing software (we used Audacity [15]), resample the audio to a sampling rate of 4096 Hz.
3. Export the audio as a signed 16-bit PCM WAVE file named “Playback.wav”.
4. Place the exported WAVE file into the same directory as AudioFileConverter.py, included in the design files. Run the script. A new file, named “FPBX.PAD” will be produced in the same directory.
5. Delete the existing “FPBX.PAD” file from the microSD card located in “PBAUD/”, and replace it with the newly generated file.

#### 5.3.3 Code compilation & upload

1. Download and install ArduinoIDE.
2. Download the design files and copy the contents of the “Software/PiedPiper/libraries” folder into the Arduino sketchbook libraries folder.
3. Open PiedPiper.ino in ArduinoIDE. Configure ArduinoIDE for use with the Adafruit Feather M4 Express, in accordance with the instructions provided by Adafruit [16].
4. Enable aggressive optimization compiler flags (–O3).
5. Use a MicroUSB cable to connect the controller to the system used to run ArduinoIDE.
6. Select the port used by the controller. Compile and upload the code.

## 6 Operation instructions

This section provides the procedure for setting up the trap in the field. Because this procedure involves opening the waterproof case of the device, it cannot be performed when it is actively raining or otherwise excessively wet in the area of deployment.

### 6.1 Deployment

1. Ensure that the trap’s battery is fully charged, and that a green light shines on the solar charge controller. If the green light is not shining, press the BOOT button to enable the charge controller. The battery can be charged by connecting the charge controller to a USB power source using a microUSB cable.
2. Find a host plant to affix the trap to. The plant must have branches or a stem that is between 0.5 cm-2 cm in diameter to serve as the substrate.
3. Install a right-angle stake or similar mounting device next to the plant, such that the trap can be hung next to the plant in the manner shown in Fig. 1.
4. Assemble the imaging box. Open the trap and connect the microUSB cable between the controller and solar charge controller to power it on. The trap will undergo a routine to test its subsystems; during this routine, the illumination LEDs should flash three times before stopping. If the LEDs begin flashing continuously, a problem has occurred.
5. Close the trap, install it into the imaging box, and place the imaging box onto the stake.
6. Feed the substrate into the imaging box such that it intercepts the camera’s field of vision. Firmly loop a piece of vibration exciter wire around the vibration exciter loop, and wrap the other end of the wire around the substrate.
7. Affix the contact microphone to the substrate. This is performed using an elastic band:
  a. Insert the microphone into the elastic band.
  b. Position the microphone on the substrate, near where the vibration exciter wire is attached.
  c. Pull one side of the elastic band over the substrate, and attach this side to the opposite end of the microphone.
8. Leave the device to collect data for up to three months, depending on the availability of solar power.

### 6.2 Retrieval and data collection

1. Dismantle the deployed trap by disconnecting it from the substrate and removing it from the imaging box. Open the case and disconnect the microUSB cable.
2. Extract the microSD card from the Hypnos board. Copy the contents of the SD card onto a personal computer.
3. Place the data parser script into the same directory that all the data was just copied to, and execute it. Three new folders should immediately appear in the directory, named “Detection Audio”, “Spectrograms”, and “Processed Spectrograms”, along with one new file named “DetectionTimes.txt”. Wait for the data parser to finish executing; this may take some time if many detections were recorded during the deployment.
4. When the parser terminates, all the data that was recorded during the deployment will be in human-readable form.
  a. The folder “Detection Audio” will contain.wav audio files, each of which will contain the audio responsible for each mating call detection. These names of these files will be the detection number that the audio in each corresponds to.
  b. The folder “Spectrograms” will contain raw spectrogram images of the audio responsible for each detection. Similarly to the audio files, the name each spectrogram will be the detection number that it corresponds to.
  c. The folder “Processed Spectrograms” will contain processed spectrogram images of the audio responsible for each detection. These spectrograms are based on the audio data responsible for each detection after it has gone through the signal processing detailed in section 2.1.2.
  d. The file “DetectionTimes.txt” contains the date and time of each positive detection, where the line number of each time corresponds to the detection number.

## 7 Validation and characterization

### 7.1 Waterproofing

The Pied Piper trap meets the requirements for IP67 rating (as laid out in the amended IEC60529 standard) for resistance to dust and water intrusion. The waterproofing of the device was validated in both a controlled indoor test and in field tests.

The controlled test consisted of verifying that no harmful levels of water intrusion into the trap occurred after immersing it under water, such that the highest point of the waterproof case was 20 cm below the waterline, for two hours. After the device was removed from the water, its exterior was manually dried using a rag before the waterproof case was opened, and its interior surfaces visually inspected to confirm that no visible amount of water had entered it. During this inspection, particular attention was paid to the possible ingress points in the waterproof case that result from the modifications made to it, including all the areas where holes are drilled into the case and sealed in subsections 5.3 and 5.4 of the Build instructions section.

The field test consisted of deploying the trap for an extended period outdoors under extremely wet conditions. The trap was placed under a small oak tree sapling (which was insufficient to provide much shielding from precipitation) in mid-February 2022 at the Oak Creek Center for Urban Horticulture and left to be exposed to a variety of weather conditions. These conditions primarily consisted of intermittent rain and overcast, and included some minor flooding. The trap was retrieved after two weeks and tested indoors to verify that it was still functional. Its internals were thoroughly inspected for signs of water damage, including corrosion on electrical components, visible condensation, and damaged adhesives. No signs of water intrusion were found.

### 7.2 Power budget

The following table provides information on the average power consumption and expected battery life of the Pied Piper trap. The power consumption of each component and subsystem of the device was determined experimentally by measuring the average operational current draw of each component at a known supply voltage. The microcontroller’s processor uses a 3.3 V logic voltage, however its supply voltage is effectively 5 V because the controller receives 5 V power form a microUSB cable and performs on-board conversion to 3.3 V (Fig. 9, Fig. 10, Fig. 11, Fig. 12, Fig. 13, Fig. 14, Fig. 15, Fig. 16, Fig. 17, Fig. 18, Fig. 19, Fig. 20).

### 7.3 Audio output

**Fig. 21:**
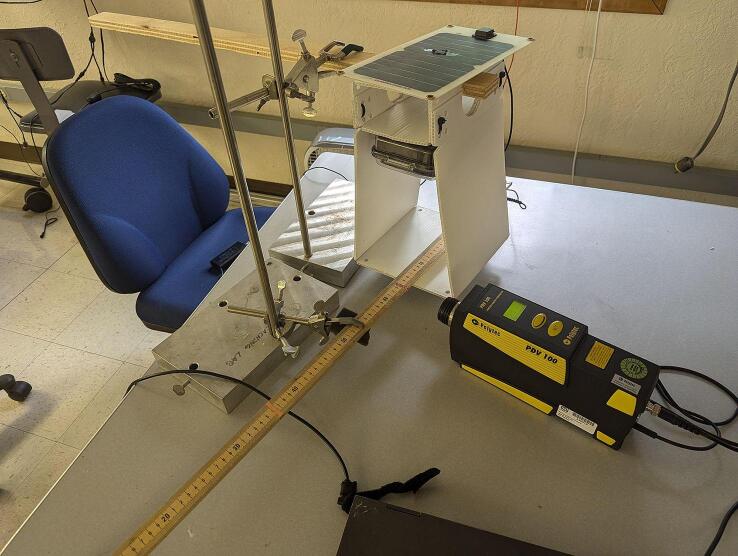
Experimental apparatus used to measure the frequency response of the audio output system. The trap and artificial substrate (meter stick) are suspended using laboratory stands in a manner consistent with the trap’s field mounting conditions.

The frequency response of the Audio output system was measured by configuring the trap to excite a meter stick (used as an artificial substrate) with an eight-second long constant amplitude linear sinewave chirp spanning 100 Hz to 1500 Hz, and a Polytec PDV-100 laser vibrometer was used to measure the induced vibration in the substrate. The experimental setup is shown in Fig. 21. The frequency response of the audio output system was computed from the vibrometer data after correcting for resonances in the substrate, and is presented in Fig. 8.

**Fig. 22:**
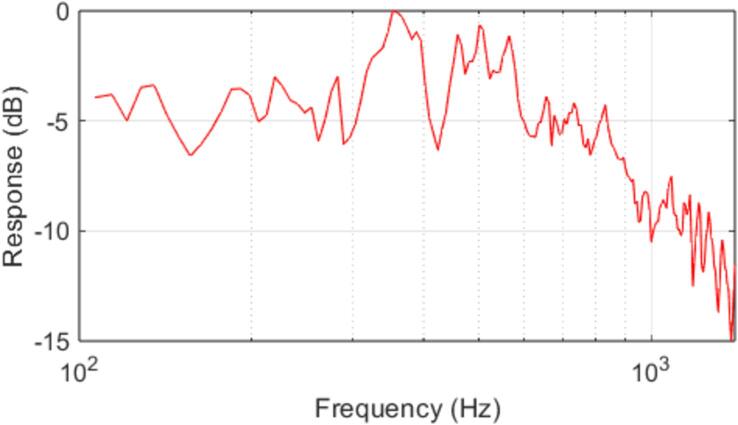
Measured frequency response of the Pied Piper audio output system after correction for substrate resonances.

A Polytec PDV-100 laser vibrometer is used to measure the vibrations induced by the trap in the substrate as shown in Fig. 22.

The frequency response of the traps was found to be sufficiently flat below 300 Hz (the region containing the majority of the spectral energy of a female *S. basalis* mating call) to avoid severely distorting the reproduced mating call, although frequency compensation of the input audio may be necessary to reproduce signals with greater energy carried by higher frequencies.

### 7.4 Field testing

Between August 26th and October 7th of 2022, nine traps were deployed to three separate vineyards that were suspected or known to harbor treehopper infestation; these vineyards will be referred to using the letters A, B, and C. The traps were positioned in peripheral areas of the vineyards near grapevines displaying symptoms of Grapevine Red Blotch Disease. All the traps were attached to plants that treehoppers are known to feed on, which primarily consisted of grape vines, apple trees, and oak trees. Throughout each deployment period, the traps were periodically serviced to retrieve data and verify their continued functionality. The data collected by the traps consisted of the times, dates, and audio associated with positive detections of treehopper mating calls, and images collected by the trap's camera modules. Detection data collected by the traps was manually reviewed by examining the spectrograms of the recorded audio and verifying the presence of a well-defined tone that gradually increases in frequency over the course of five to eight seconds; this manual review process was conducted to assess the efficacy of the detection algorithm under field deployment conditions, and is not strictly necessary for the operation of the traps. Any data that did not have the characteristic tone was discarded. During this deployment, a low value of the amplitude significance threshold (SIG_THRESH = 3.5 in PiedPiper.h) was used to minimize its false negative detection rate, which came at the expense of increasing its false positive rate.

**Fig. 23:**
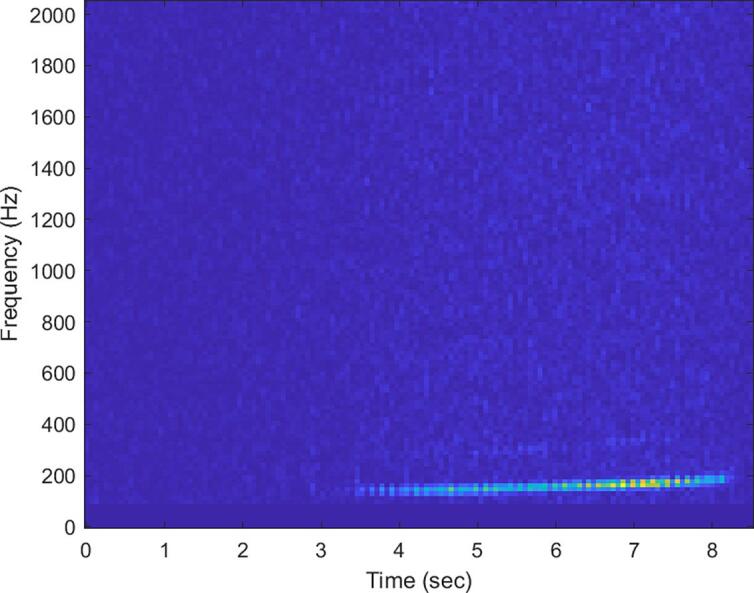
Spectrogram of a likely *Stictocephala basalis* mating call recorded from Vineyard B on August 25th, 2022 at 4:05:33 pm.

**Fig. 24:**
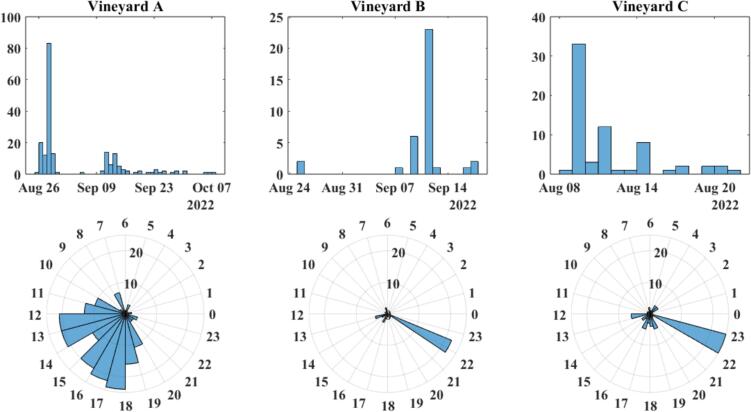
Daily and hourly distribution of positive mating call *Stictocephala basalis* detections recorded by Pied Piper traps deployed to vineyards A, B, and C.

**Fig. 25:**
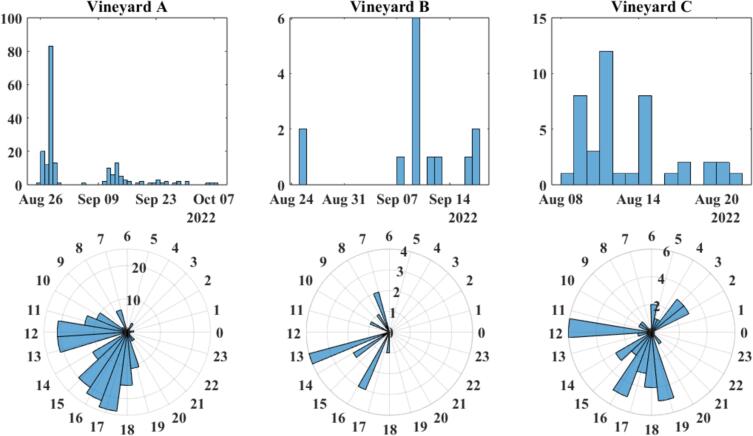
Daily and hourly distribution of positive mating call *Stictocephala basalis* detections recorded by Pied Piper traps, with all detections at 22 h removed.

During each respective deployment period, a total of 195, 36, and 67 positive detections were recorded at vineyards A, B, and C respectively. A spectrogram of one of these positive detections is presented in Fig. 23, and the temporal distributions of detection data recorded by the traps are presented in Fig. 24. The data appears to show the overall frequency of positive detections decreasing as the season progresses into Fall. The average hourly distribution of detections is similar between Vineyards B and C, but significantly different from Vineyard A, and this is because the data collected from both B and C are skewed by a large string of positive detections occurring around 2:00 am. While this is interesting and important information, this string of detection was outside of the normal observed behavior. Omitting these detections from the data collected from Vineyards B and C results in hourly distributions more consistent with that seen from Vineyard A; this is visualized in Fig. 25. This is not intended to invalidate or diminish the significance of 2:00 am detections however.

**Fig. 26:**
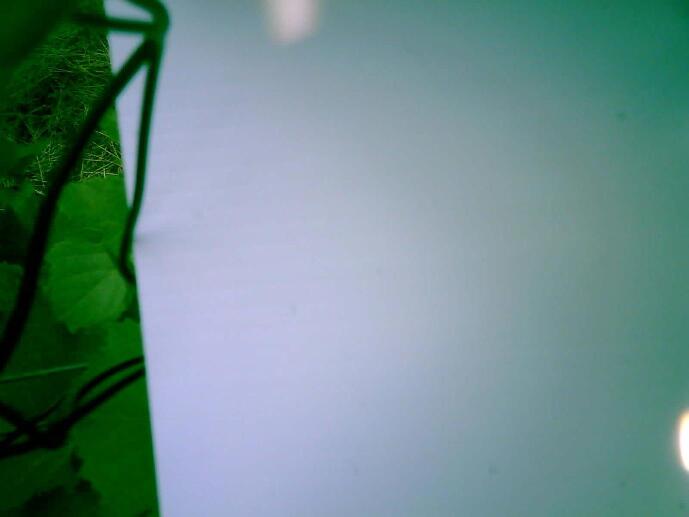
Image captured by a Pied Piper trap showing no visible signs of a treehopper despite a positive acoustic detection.

**Fig. 27:**
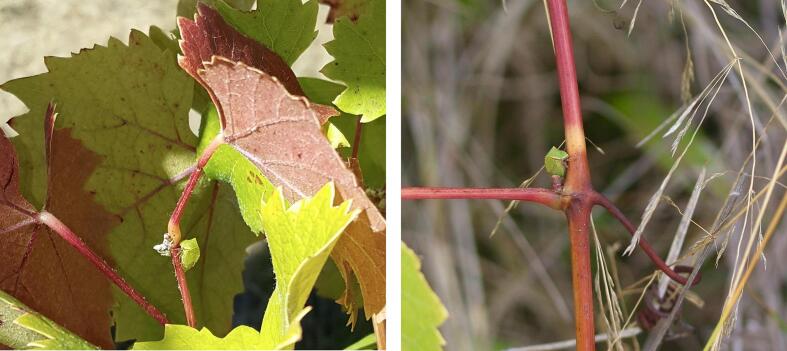
Visual sightings of *S. basalis* treehoppers at Vineyard B.

None of the traps captured conclusive photos of treehopper specimens responsible for the positive detections, and this is likely due to a problem with the trap’s ability to lure the insects. Fig. 26 provides an image taken by a trap at Vineyard B associated with the acoustically detected mating call shown in Fig. 27, showing that no treehoppers are visible in frame. Most of the traps were deployed such that the substrate was out of frame of the camera, with only the vibration exciter wire was visible; in this configuration, it was for insects to traverse the substrate and begin climbing the wire towards the source of the vibration, where it would then be visible to the camera. This arrangement is more ergonomic for the user when deploying the traps, however it also prevents the camera from imaging insects if they do not choose to climb the wire. Several different hypotheses for why the insects did not choose to traverse the wire are:

● The insects were adverse to climbing on an unnatural substrate.
● Something is wrong with the signal being outputted by the vibration exciter, or the high rigidity of the wire prevented the insects from using their normal navigational abilities.
● The insects detected were not treehoppers, and were instead some other species that produce a vibrational signal similar to that of a male treehopper’s mating call.
● The playback signal produced by the traps was severely distorted by uneven frequency response of the substrate, as this is a known challenge with vibrational signal reproduction [17] that the traps currently do not address.

Despite the lack of photographic verification, it is unlikely that the detected signals originated from sources other than male treehopper mating calls. Prior to the deployment, treehoppers were visually spotted at Vineyard B (see Fig. 26), and the characteristics of the detected signals do not precisely match those of any known environmental noise or signals from other insect species. There is an elevated chance that some of the signals detected at Vineyard A were false positives, as this location exhibits a significantly different temporal distribution of mating calls to the other two vineyards, which may suggest the presence of a different species that produces a signal similar to that of a male *Stictocephala basalis* mating call.

## 8 Future direction

The Pied Piper traps have successfully demonstrated acoustic surveillance of agricultural pest insects. However, the have not yet been used to collect data on a large scale for an entomological study. Additionally, some aspects of the traps’ performance in the field is poorly understood, particularly their lack of success in imaging acoustically detected treehoppers. This indicates that additional laboratory testing is needed to investigate the process of luring and imaging detected specimens. Finally, the traps presented here are specialized for monitoring only a single insect species; a more generalized detection algorithm capable of classifying different signals is necessary to enable them to monitor multiple species. A future detection algorithm based on machine learning may prove to be more robust than the relatively simple frequency domain analysis used in the current version, however the implementation of such an algorithm would necessarily require a database of many different samples of mating calls of each target species, and poses challenges for implementation on a low-cost, low-power microcontroller platform.

In a practical setting, the data collected by a large quantity of deployed Pied Piper traps may be useful for informing the use of integrated pest control measures. Additionally, the traps may be made more directly useful for reducing populations of pest insects by deploying them alongside typical adhesive or container traps.

## CRediT authorship contribution statement

**Vincent Vaughn:** Writing – original draft, Validation, Software, Methodology, Investigation. **Andrew Ensinger:** Writing – review & editing, Validation, Investigation. **Edwin Harris:** Writing – original draft, Validation, Resources. **Elijah Shumway:** Methodology, Investigation, Conceptualization. **Rachele Nieri:** Writing – review & editing, Data curation, Conceptualization. **Vaughn Walton:** Writing – review & editing, Validation, Resources, Funding acquisition, Conceptualization. **John Selker:** Writing – review & editing, Resources, Funding acquisition. **Chet Udell:** Writing – review & editing, Supervision, Resources, Investigation, Funding acquisition, Conceptualization.

## Declaration of competing interest

The authors declare that they have no known competing financial interests or personal relationships that could have appeared to influence the work reported in this paper.
