# Supplementary material for: Open-source insect camera trap with vibrational detection and luring for monitoring Stictocephala basalis (Walker, Hemiptera: Membracidae: Smiliinae)
Source: HardwareX. 2024 Nov 15;20:e00604. doi: 10.1016/j.ohx.2024.e00604 (PMC11626055; doi:10.1016/j.ohx.2024.e00604)
Supplement: Supplementary Data 1 [file mmc1.pdf]

Pied Piper Design Files

Design files for Pied Piper can be found on Zenodo:
https://zenodo.org/records/10810501
